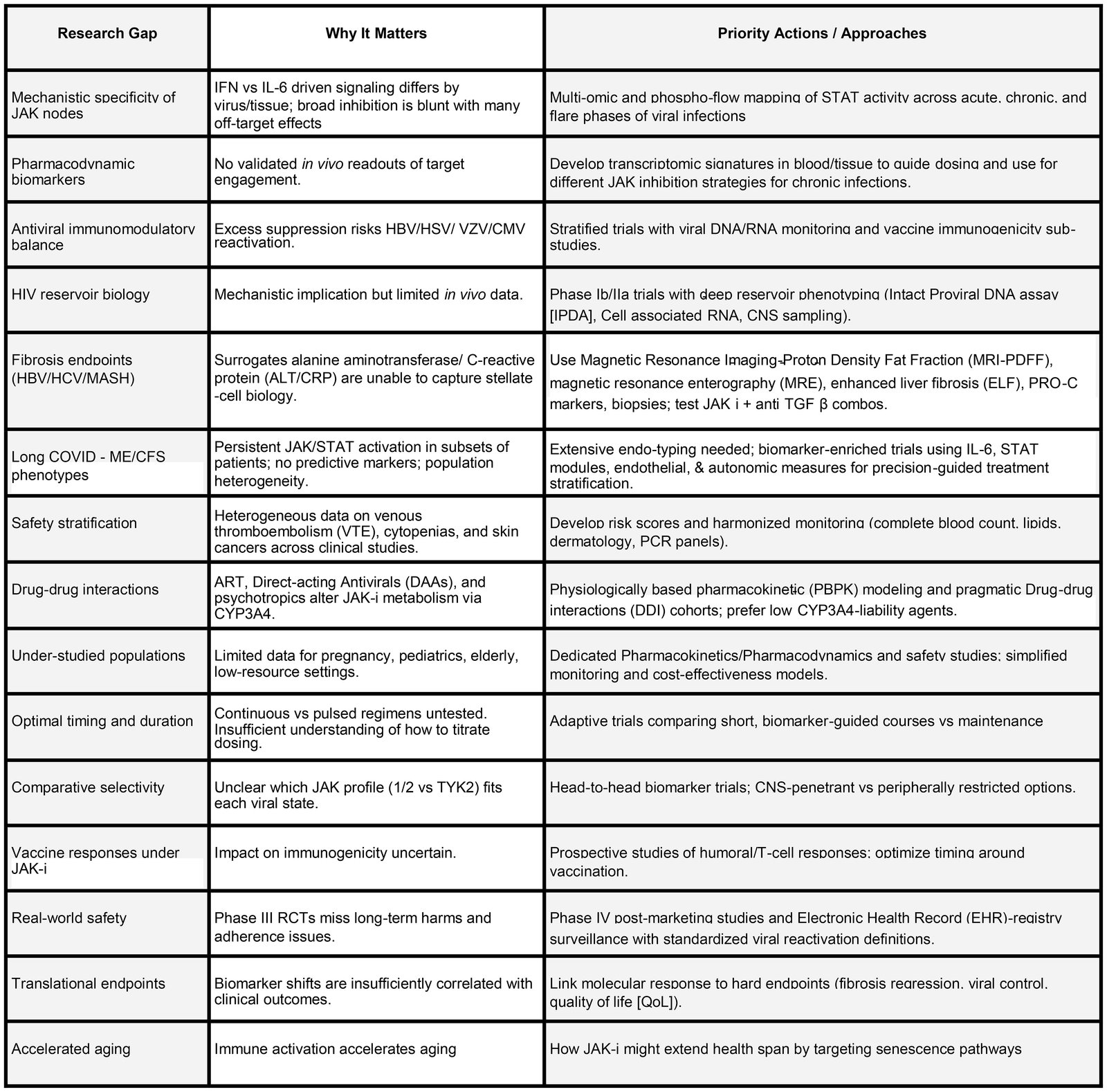# Targeting the JAK/STAT pathway in chronic viral infections: opportunities and challenges for immunomodulation

**DOI:** 10.3389/fimmu.2026.1887876

**Published:** 2026-08-21

**Authors:** Nathalie Garnier, Julia Ossi, Vincent C. Marconi, Raymond F. Schinazi, Boghuma K. Titanji

**Affiliations:** 1 Center for ViroScience and Cure, Laboratory of Biochemical Pharmacology, Department of Pediatrics, Emory University School of Medicine and Children’s Healthcare of Atlanta, Atlanta, GA, United States; 2 Department of Medicine, Emory University School of Medicine, Atlanta, GA, United States; 3 Division of Infectious Diseases, Emory University School of Medicine, Atlanta, GA, United States; 4 Department of Global Health, Rollins School of Public Health, Atlanta, GA, United States; 5 Emory Vaccine Center, Emory University, Atlanta, GA, United States; 6 The Joseph Maxwell Cleland Atlanta VA Medical Center, The Atlanta Veterans Affairs Medical Center, Decatur, GA, United States

**Keywords:** chronic viral infection, clinical studies, herpesviruses, HIV-1, JAK inhibitor, long covid, SARS-CoV-2, hepatitis viruses

## Abstract

The discovery of the Janus kinase/signal transducer and activator of transcription (JAK/STAT) pathway as a key regulator of immune function and inflammation led to the development of JAK inhibitors (JAK-i), several of which are now approved for autoimmune and chronic inflammatory conditions. Recently, JAK-i have been repurposed for infectious diseases, most notably with baricitinib, a United States Food and Drug Administration (FDA)-approved JAK1/2 inhibitor for individuals hospitalized for COVID-19, demonstrating a survival benefit by dampening cytokine-mediated hyperinflammation. This has generated increased interest in leveraging JAK-i for treating people with viral infections associated with chronic immune activation, inflammation or viral persistence. This review summarizes JAK/STAT signaling in viral infections characterized by latency or chronic sequelae. We explore how modulation of this pathway may alter immune responses, impact viral control, and mitigate the consequences of chronic inflammation. We also critically examine the therapeutic potential and limitations of JAK-i in these contexts. Finally, we outline key gaps in the field and propose future directions for research into JAK/STAT targeted immunomodulation in chronic viral infections.

## 1 Introduction

**Figure 1:**
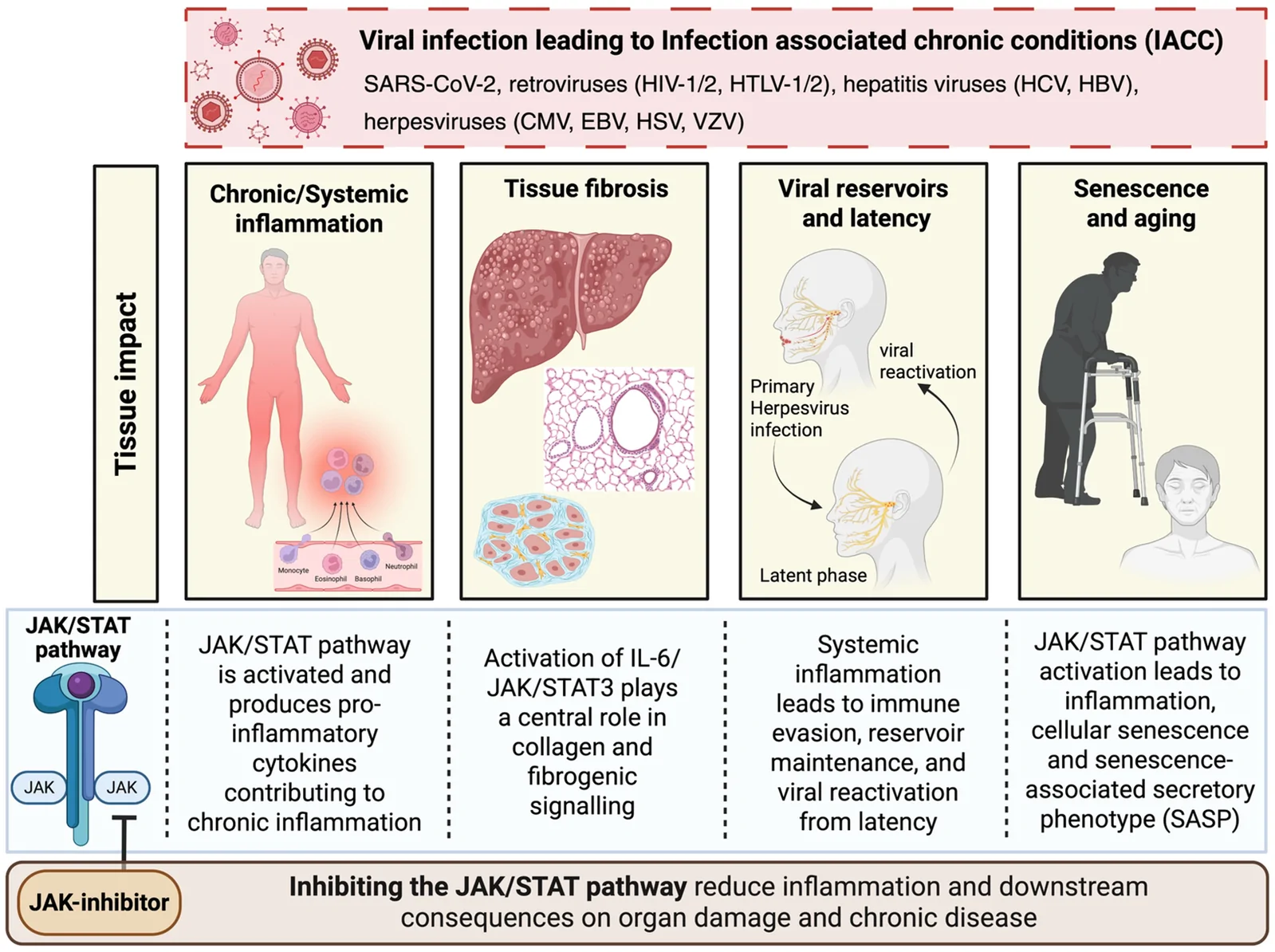
Leveraging JAK- STAT inhibition to mitigate virus induced chronic disease manifestations. Created in https://BioRender.com.

Acute viral illnesses cause immediate morbidity and infrequent mortality, though, in some instances, their long-term impact can extend beyond the acute phase of infection, manifesting as chronic disease with profound and often debilitating consequences for host health. Individuals who develop chronic manifestations of viral infections can have rapidly progressive neurocognitive impairment, functionally limiting fatigue or exercise intolerance, or even severe peripheral nerve and joint pain. Collectively, these overlapping clinical manifestations are termed infection-associated chronic conditions (IACC), which are especially prominent in viruses that establish lifelong persistence through latency or chronic active replication. Key examples include human retroviruses like human immunodeficiency virus type 1/2 (HIV-1/2) and human T lymphotropic virus type 1 (HTLV-1); herpesviruses including human cytomegalovirus (CMV), varicella zoster virus (VZV), herpes simplex virus 1/2 (HSV-1/2), and Epstein-Barr virus (EBV); chronic hepatitis viruses like hepatitis C, B and D viruses (HCV, HBV and HDV); as well as viruses capable of driving post-viral syndromes through persistence of viral antigen and genetic material or sustained immune activation (e.g. severe acute respiratory syndrome coronavirus [SARS-CoV-2]). The pathological sequelae of these chronic infections are closely tied to immune dysregulation and persistent inflammation (**Figure 1**). A growing body of evidence suggests that immunomodulatory therapies could play a critical role in mitigating these downstream effects. Among potential targets, the Janus kinase/signal transducer and activator of transcription (JAK/STAT) pathway is a key regulator of inflammatory responses in chronic viral infections. Pharmacologic modulation with small-molecule JAK inhibitors (JAK-i) offers a promising strategy to attenuate immune-mediated damage while preserving essential antiviral defenses. In this review, we examine the role of JAK/STAT signaling in persistent viral infections and their clinical manifestations, highlighting both the opportunities and the challenges of leveraging JAK-inhibition as a therapeutic approach to address chronic viral infections.

## 2 The role of the JAK/STAT pathway in immune response and inflammation

The JAK/STAT pathway is a highly conserved signal transduction pathway that is important for immune regulation by mediating cytokine responses and orchestrating both innate and adaptive immunity (1, 2). In the canonical JAK/STAT signaling pathway (**Supplementary Figure 1****),** activation begins when a ligand binds its receptor; over 50 cytokines, hormones and growth factors act as ligands. JAK is a family of intracellular proteins that mediate cytokine receptor signaling; JAK1, JAK2, JAK3 and TYK2, each of which has specific functions. STATs are cytoplasmic transcription factors which dimerize and translocate to the nucleus to regulate transcription of selected genes. The genes transcribed in response to STATs are mostly involved in immune regulation, inflammation including several of the same cytokines that triggered their activation. This creates a positive feedback amplification loop promoting cellular proliferation, and apoptosis depending on the environmental, immunological stimuli and the STAT that is phosphorylated (STAT1, STAT2, STAT3, STAT4, STAT5a, STAT5b and STAT6).

To prevent pathological inflammation and autoimmunity triggered by the pathway itself, JAK/STAT is regulated by a range of negative feedback mechanisms, including suppressors of cytokine signaling (SOCS) (1, 2). Mostly, SOCS1, SOCS2, SOCS3 and CIS inactivate JAK proteins, preventing STAT from reaching the receptor binding site, and blocking the access of signaling proteins to the proteasome.

Furthermore, disruption or mutations in the JAK/STAT pathway can cause dysregulation of cell growth or immunologic disorders and have been associated with various diseases, including autoimmune syndromes, hematological malignancies, and chronic viral infections (1, 2). Small molecule synthetic drugs known as JAK-i have been developed to target a variety of autoimmune, inflammatory and hematological disorders (3). Currently, 11 JAK-i are approved by United States Food and Drug Administration (FDA) for various disorders, with several under study in clinical trials (4).

The use of JAK-i in the treatment of immune dysregulation caused by infectious disease pathogens is growing. For example, our group conducted a randomized controlled trial (RCT) of ruxolitinib (5) to reduce systemic inflammation and HIV persistence in people with HIV (PWH) on antiretroviral therapy (ART). Also, during the coronavirus disease 2019 (COVID-19) pandemic, JAK-i rapidly emerged as an immunomodulatory class that could be used to target the cytokine storm in severe COVID-19 (6, 7). Two large RCT RECOVERY (8) and COV-BARRIER (9) demonstrated a significant mortality benefit in patients with severe COVID-19 who were treated with baricitinib, having a similar safety profile to standard of care. A systematic review found that JAK-i reduced mortality, independent of dexamethasone or tocilizumab, and probably decreased adverse events compared to patients who were treated without a JAK-i (10). The successful and safe use of JAK-i in critically ill patients with severe COVID-19 has opened the door for exploring their utility in inflammation and immune mediated manifestations of other viral infections.

## 3 JAK/STAT pathway: pharmacologic targeting

JAK-i are broadly divided in two categories: first-generation and the newer/next generation drugs (1). First-generation JAK-I act through competitive inhibition by forming non-covalent interactions with the ATP-binding site in the highly conserved JH1 kinase domain of JAK. This allows targeting of multiple JAK members with low specificity, increasing the probability of off-target and adverse effects. The next generation is more specific for JAK members and more selective in targeting preferentially a particular JAK isoform over other JAKs, improving tolerability with fewer off-target and adverse effects.

As highlighted in the literature (11–13), JAK-i have immunomodulatory properties in chronic diseases but also cause immunosuppression, which may increase the risk of certain infections as reactivation of herpesviruses e.g. VZV (**Supplementary Table 1**). Although JAK-i are relatively safe and well tolerated, they carry a black box warning label for increased risk of serious heart-related events, cancer and death from the FDA, depending on the clinical context and individual patient characteristics (e.g., age and medical history). This variability underscores the necessity of developing more tailored, individualized therapeutic approaches that also consider the viral context.

## 4 JAK/STAT signaling in chronic viral infections

### 4.1 HIV-1 and HTLV-1

HIV-1 causes acquired immunodeficiency syndrome (AIDS) characterized by immune dysfunction, immune deficiency and increased susceptibility to opportunistic pathogens (14). This is mostly due to the depletion of immune cells which carry the CD4 receptor and the viral co-receptors CCR5 and CXCR4 (CD4+ T-cells, macrophages and dendritic cells) by HIV during acute infection (14, 15). Afterwards, viral replication is sustained by induced systemic chronic inflammation and immune activation, even with ART (7).

Among the cellular pathways affected by HIV-1, the JAK/STAT pathway is targeted in multiple ways to promote immune evasion and enhance viral replication and persistence (14). Although viral mechanisms affecting the JAK/STAT pathway and the precise roles of individual STAT proteins are still incompletely understood, accessory virus proteins targeting different STATs have been identified (**Supplementary Table 2**). STAT1, STAT3 and STAT5 are impacted by HIV-1 in complex, context- and time-dependent ways across different cellular environments. These alterations persist in patients on ART, despite CD4+ T-cell recovery and may contribute to immuno-aging in PWH. This is further exacerbated by chronic inflammation fueled by JAK/STAT pathway-induced inflammatory cytokines (interleukin [IL]-1, IL-6, IL-10 and interferon [IFN]-α and γ). Chronic inflammation in HIV infection is linked to increased risk of chronic end organ diseases, as well as morbidity and mortality, in both treated and untreated PWH (14). Additionally, the JAK/STAT pathway regulates the cellular environment required for HIV-1 persistence by modulating CCR5 and CCR2 expression (involved in HIV-1 reservoir maintenance and immune cell recruitment) (16, 17).

In recent years, clinical evidence to support these *in vitro* observations has emerged. For example, Saez-Cirion et al. noted sustained viral remission off ART in a patient following allogenic hematopoietic stem cell transplantation (HSCT) and a prolonged course of ruxolitinib and corticosteroids to treat recurrent graft versus host disease (GVHD) post-transplantation (18). Unlike previous PWH who have achieved sustained remission following HSCT, this individual received stem cells from a wild-type CCR5 donor, so remained susceptible to HIV after transplantation. Both Allo-HSCT and GVHD reduce the viral reservoir; therefore, the conditioning regimen and repeated GVHD in this individual may have contributed to its elimination. The immunosuppressive environment caused by ruxolitinib therapy may also have contributed, given the ability of ruxolitinib to block HIV replication, viral reactivation and reservoir reseeding, as demonstrated in published *in vitro* and *ex vivo* studies (18). Additionally, a phase 2 open-label RCT led by our group studied ruxolitinib in PWH on ART and showed that not only was the drug well tolerated; it also reduced levels of B-cell lymphoma-2 (BCL-2), a marker of cell survival. This down-regulation of BCL-2 in HIV-infected cells may tip the balance in these long-lived cells towards apoptosis, further reducing the viral reservoir (5, 19).

These promising data regarding JAK-i in the context of HIV persistence have paved the way for clinical trials that are currently studying its effects as a component of HIV functional cure strategies in PWH on ART. One Phase II trial of baricitinib (20) is targeting HIV reservoirs in the central nervous system (21) and ART treatment interruption (22). Outside of the HIV cure and reservoir targeting space, JAK-i are also being explored for non-AIDS related chronic conditions mediated by inflammation in PWH including mood disorders (23).

HTLV-1 is another retrovirus which causes chronic lifelong infection in humans. While most cases of infection are asymptomatic, chronic infections increase the lifetime risk for adult T cell leukemia. Interleukin-2 receptor alpha (IL2Rα) is expressed in cancer cells of adult T cell leukemia leading to activation of the JAK/STAT pathway and spontaneous proliferation. Recent studies have shown that JAK1 inhibitors combined with mTORC1/C2 inhibitors led to decreased proliferation of cancer cells in ATL associated with HTLV-1 infection (24).

### 4.2 SARS-CoV-2 and long COVID

SARS-CoV-2, responsible for COVID-19, primarily infects the respiratory system but can progress to severe multi-organ dysfunction (25). Severe cases occur when the innate immune response is inadequate to control viral replication. This triggers a dysregulated immune response, which is marked by acute respiratory distress syndrome (ARDS) and systemic hyperinflammation. Systemic hyperinflammation is characterized by uncontrolled and high levels of proinflammatory cytokines and chemokines like IL-6, IL-10, IL-21, IFN-γ, and tumor necrosis factor (TNF)-α leading to tissue damage and multi-organ failure (26). Beyond the acute phase of infection, long-term effects of COVID-19 remain a serious complication in about 5-10% of individuals. This cluster of persistent chronic symptoms, which fail to resolve following acute infection, has been termed “Long COVID” (LC). Clinical manifestations include neurologic, gastrointestinal, pulmonary, and cardiovascular symptoms, leading to significant disability and morbidity in those affected (27, 28). Several mechanisms are being investigated including persistent viral infection, latent virus reactivation, immune dysregulation, autoimmunity, gut microbiota changes, endothelial injury and coagulation abnormalities (27–29).

Some viral proteins of SARS-CoV-2 target the JAK/STAT pathway as an immune escape strategy by inhibiting IFN receptors or directly targeting STAT1 and STAT2 (30) (**Supplementary Table 2**). Also, the systemic hyperinflammation of severe acute COVID-19 impacts the JAK/STAT pathway through the overexpression of cytokines, chemokines, and interferons. In severe acute COVID-19, alongside standard treatment such as corticosteroids and antivirals, baricitinib effectively reduces mortality in hospitalized patients (8, 9). By contrast, two other clinical trials, phase 3 RUXCOVID (31) and phase 4 TACTIC-R (32) evaluating JAK-i in patients with moderate-to-severe COVID-19 concluded that ruxolitinib and baricitinib, respectively, showed no significant efficacy. Differences in treatment duration and sample size, as well as the early use of dexamethasone (which masked the additional effect of the anti-JAK drug), may explain the discrepancy observed between the TACTIC-R and COV-BARRIER study.

Analysis of peripheral blood mononuclear cells showed upregulation and persistent activation of the JAK/STAT pathway in affected individuals a median of 28 months after infection, suggesting that chronic inflammation mediated by this pathway is a likely contributor to the pathogenesis of LC (33). Given these findings, JAK-i are being studied as potential therapeutic agents for treating individuals with LC (34–36). An ongoing phase 3 clinical RCT aims to determine whether baricitinib can improve neurocognitive impairment and exercise intolerance among patients with LC.

### 4.3 HBV, HDV, and HCV

HBV and HCV infections are responsible for chronic liver disease and increase the risk for developing hepatocellular carcinoma (HCC) (37). Both are associated with persistent inflammation, as evidenced by elevated IL-6 levels in the serum of infected patients. Both viruses affect the JAK/STAT pathway by continuously activating it *via* elevated IL-6 levels that bind the gp130 receptor, promote JAK phosphorylation, and activating STAT3 (38) as well as SOCS (39) (**Supplementary Table 2**). It is widely assumed that this promotes viral replication and the progression to HCC.

Co-infection with HBV and HDV leads to the most aggressive form of chronic viral hepatitis. JAK1 has been shown to exhibit proviral activity *in vitro* during HDV infection, making JAK1 inhibitors like Upadacitinib which is FDA-approved interesting novel therapeutic options worthy of further investigation (40).

In chronic hepatitis infections, persistent inflammation drives liver fibrosis activating hepatic stellate cells *via* cytokine signaling. The IL-6/JAK/STAT3 pathway plays a central role in sustaining collagen deposition and amplifying fibrogenic signaling through crosstalk with TGF-β. Preclinical studies in non-viral models of liver fibrosis demonstrate that JAK-inhibition can suppress hepatic stellate cell activation and reduce fibrotic markers; for example, pharmacologic JAK2 antagonism attenuated hepatic fibrogenesis and collagen deposition *in vivo* (41, 42). These findings have prompted interest in JAK-i as potential anti-fibrotic agents in metabolic dysfunction–associated steatohepatitis (MASH), a condition that shares several pathogenic pathways with fibrosis observed in chronic viral hepatitis (43). However, in the context of viral hepatitis, targeting JAK signaling represents a double-edged sword. JAK-i use has been associated with reactivation of hepatitis B in patients not receiving antiviral therapy (44). While JAK-inhibition offers a mechanistically plausible strategy to attenuate fibrosis, the underlying JAK isoform dependencies differ meaningfully between the fibrogenic and antiviral arms of this pathway. Fibrogenic hepatic stellate cells activation is driven predominantly through JAK2/STAT3 downstream of IL-6, with an additional JAK1-dependent contribution via TGF-β/STAT3 crosstalk (45, 46). By contrast, host antiviral defense against HBV and HCV depends on JAK1 and TYK2 signaling downstream of type I (IFN-α/β) and type III (IFN-λ) interferons: both interferon classes require JAK1, while type I signaling additionally requires TYK2 (47, 48). Because JAK1 sits at the convergence point of both antiviral IFN signaling and part of the fibrogenic TGF-β/STAT3 axis, broad-spectrum JAK-inhibition cannot cleanly separate these effects. However, this isoform asymmetry raises the possibility that next-generation, more selective JAK2 inhibitors, which largely spare JAK1 might attenuate IL-6-driven fibrogenic signaling while more substantially preserving JAK1/TYK2-dependent antiviral IFN responses than pan-JAK or JAK1/2 dual inhibitors currently used in clinical practice.

### 4.4 Herpesviruses (EBV, CMV, HSV)

Herpesviruses establish lifelong latent infections that can reactivate, persisting throughout the host’s life. Reactivation is triggered mainly by stressors and immunosuppressive states (49). Here, we will focus on three of these viruses: HSV-1, CMV, and EBV. Each of these viruses establishes latency in different host compartments. Viral latency hijacks the cellular machinery to promote cell survival proliferation and genetic instability which can lead to the development of pathological disease manifestations (**Supplementary Table 2**). EBV has the most complex latent infection program for persistence in resting memory B cells while being non-pathogenic and undetectable [i.e., absence of viral protein production (50)].

*In vitro* EBV treatment with tofacitinib inhibited the growth of EBV-positive T/NK lymphoma cells, induced G1 cell-cycle arrest, and inhibited the growth of established tumors in mice with immunodeficiency due to functional deficiencies in T, B, and NK cells (51). Also, JAK2 inhibitors have been tested for inducing apoptosis in lymphoma cell lines and for controlling inflammation-related clinical manifestations (52, 53). Recently, ruxolitinib was studied in a phase 2 trial for the treatment of systemic chronic active EBV disease and resulted in a disappearance of disease activity in 22.2% of the 9 patients who received the drug (54).

Some studies have linked EBV (and to a lesser extent CMV) reactivation or latent viral persistence with Myalgic Encephalomyelitis/Chronic Fatigue Syndrome (ME/CFS) (55, 56) and LC (27). A study suggests CMV may protect against neurocognitive symptoms in LC (57, 58). Targeting the JAK/STAT pathway in post-viral fatigue syndromes, including those triggered by EBV reactivation, may have some therapeutic potential. Persistent cytokine and interferon signaling can sustain immune activation and fatigue, making JAK-inhibition a biologically plausible strategy to interrupt this loop. However, evidence remains limited, and the approach which carries the risk of blunting JAK/STAT signaling could impair antiviral control and worsen viral persistence. While no trials have yet tested JAK-i in classic ME/CFS, several clinical studies are now evaluating drugs like baricitinib and ruxolitinib in LC. These trials may help clarify whether modulating JAK/STAT signaling can safely rebalance chronic immune activation without compromising antiviral defense. For now, the idea remains speculative but mechanistically intriguing and requiring rigorous clinical validation and careful patient selection before translation to ME/CFS or EBV/CMV-triggered fatigue states.

Although JAK-i are generally well tolerated, they carry an FDA boxed warning for an increasing risk of herpesvirus reactivation, specifically VZV and HSV1/2. This likely reflects the central role of JAK/STAT signaling in mediating antiviral immunity, including type I, II, and III interferon responses, as well as cytokine pathways with both pro- and anti-inflammatory functions (e.g., IL-6 and IL-10). Consequently, the immunologic impact of JAK-inhibition may vary depending on the timing of administration relative to viral exposure or infection stage. Early inhibition during acute infection and autoimmune context could theoretically impair interferon-mediated antiviral defenses, whereas in chronic viral infections or states of persistent immune activation, selective attenuation of JAK-STAT signaling may mitigate pathologic inflammation. These considerations underscore the importance of careful patient selection and preventative strategies, including vaccination, screening for VZV prior to initiation of JAK-i therapy, and antiviral prophylaxis when appropriate (60, 61). The optimal timing of vaccination relative to JAK-i administration remains an area of active investigation; for example, ongoing studies in patients with rheumatoid arthritis are evaluating optimal scheduling of recombinant zoster vaccine (RZV) in individuals receiving tofacitinib (59). For antiviral prophylaxis, agents such as acyclovir and valacyclovir, are considered for patients with low CD4 and/or prior history of reactivation (60, 61). In parallel, a structured monitoring plan should be implemented to ensure early clinical assessment for suspected reactivation signs and treat suspected VZV promptly. However, small studies have not found evidence of herpesvirus reactivation in PWH after JAK-i (62, 63). Overall, evidence in chronically infected populations remains limited, so individualized risk-benefit assessment and future studies are needed to optimize screening, prophylaxis, and monitoring strategies tailored to these groups.

## 5 Chronic viral infections, accelerated aging, and the JAK/STAT pathway

Chronic viral infections drive cellular proliferation and persistent immune activation that accelerate biological aging (64). Chronic exposure to pathogenic antigens drives expansion of immune cells leading to “replicative senescence”. This rate of replication accelerates the natural rate of cellular senescence and associated telomere shortening, which are notable hallmarks of aging (65). Additionally, continuous stimulation of cytokine networks, especially IL-6, IFN, and TNF sustain JAK/STAT signaling, fueling “inflammaging”, immune exhaustion, endothelial dysfunction, and fibrosis (64). HIV and HCV infections have been associated with telomere shortening and accelerated biological aging (65–67) and an increase in senescence markers has also been reported in people with chronic viral infections (68). Senescent cells accumulate in multiple tissues, this process can be exacerbated by chronic inflammatory states induced by viral infections. Senescent cells secrete a wide range of pro-inflammatory cytokines, chemokines, and growth factors which define senescence-associated secretory phenotype (SASP) (69). JAK-i has been shown to suppress SASP in senescent cells and by so doing could alleviate the tissue dysfunction associated with cellular senescence (69, 70). Targeting SASP with senomorphics (e.g. anti-inflammatory agents) and selectively eliminating senescent cells with senolytics (e.g. BCL-2 antagonists) are considered the most promising strategies for improving the health span in an aging population and should be tested in cohorts with individuals presenting chronic viral infection. The goal is recalibration and not broad immunosuppression. JAK/STAT inhibitors are among the senotherapeutics that possess both senomorphic and senolytic properties. Future studies pairing JAK/STAT modulation with biomarkers of aging and immune recovery will help define how controlling this pathway might extend health span in the context of chronic viral infection.

## 6 Conclusion

Across chronic viral diseases, persistent inflammation and immune dysregulation frequently converge on aberrant JAK/STAT signaling. Targeting this pathway offers a strategy to rebalance immune responses, blunt hyperinflammation, and limit tissue injury without fully undermining antiviral defenses (**Table 1**). Emerging clinical data demonstrate that this balance is achievable. Going forward, precision will be critical in defining which JAK nodes to target, in which tissues, for how long, and in which chronic viral contexts.

**Table 1:** Research gaps and priorities.