# Supplementary material for: Targeting the JAK/STAT pathway in chronic viral infections: opportunities and challenges for immunomodulation
Source: Front Immunol. 2026 Aug 21;17:1887876. doi: 10.3389/fimmu.2026.1887876 (PMC13542882; doi:10.3389/fimmu.2026.1887876)
Supplement: Supplementary file 1 [file SupplementaryFile1.docx]

Supplementary Material Figure S1

**Supplementary Figure S1: JAK/STAT pathway with viral interaction points.**

On the left is the canonical JAK/STAT pathway with its five major steps. First, the JAK/STAT pathway is initiated by the binding of a ligand to the JAK receptor. Then, JAK is phosphorylated as well as the receptor. This leads to STAT recruitment and phosphorylation. Finally, STAT dimerize and translocate into the nucleus to allow transcription of genes involved in immune regulation, inflammation, cellular proliferation, and apoptosis.

On the right are represented main viral components of the different viruses described in this paper and their impact on the JAK/STAT pathway (Table S2) which justifies the use of JAK-i to counteract their viral effects on host system. The red and green arrows represent the respective inhibition and activation of the JAK/STAT pathway caused by the virus. The dotted arrows represent indirect interactions by viral proteins, and the solid arrows represent direct interactions by viral proteins. The big arrows represent the impact on viral infection. Created in https://BioRender.com
