# Supplementary material for: Targeting the JAK/STAT pathway in chronic viral infections: opportunities and challenges for immunomodulation
Source: Front Immunol. 2026 Aug 21;17:1887876. doi: 10.3389/fimmu.2026.1887876 (PMC13542882; doi:10.3389/fimmu.2026.1887876)
Supplement: Supplementary file 2 [file Table1.docx]

Supplementary Material Table S1

|  | Pacritinib | Ruxolitinib | Baricitinib |
| --- | --- | --- | --- |
| Approved indication | | | |
| Rheumatoid arthritis (1) |  |  | **X** |
| Myelofibrosis (2) | **X** | **X** |  |
| Polycythemia vera (2) |  | **X** |  |
| Severe Covid-19 (3,4) |  |  | **X** |
| Graft-versus-host disease (5) |  | **X** |  |
| Studied indication (not yet approved) | | | |
| HIV-1 cure (5–7) | **X** | **X** | **X** |
| Hepatic fibrosis (8,9) |  | **X** | **X** |
| Long COVID (10) |  |  | **X** |
| Adverse effects | | | |
| VZV reactivation (2) | **X** | **X** | **X** |
| Cytopenias (2) | **X** | **X** | **X** |
| Venous Thromboembolism (2) |  | **X** | **X** |
| Non-melanoma skin cancers (2) | **X** | **X** | **X** |

**Supplementary Table S1: Approved JAK inhibitors and profiles, with focus on infectious diseases^[1]^.**

**References for Table S1**

1.	Fraenkel L, Bathon JM, England BR, St.Clair EW, Arayssi T, Carandang K, Deane KD, Genovese M, Huston KK, Kerr G, et al. 2021 American College of Rheumatology Guideline for the Treatment of Rheumatoid Arthritis. *Arthritis Care & Research* (2021) 73:924–939. doi: 10.1002/acr.24596

2.	NCCN Clinical Practice Guidelines in Oncology: myeloproliferative neoplasms. *National Comprehensive Cancer Network* (2025) https://www.nccn.org/guidelines/guidelines-detail?category=1&id=1477 [Accessed October 10, 2025]

3.	Spinelli FR, Meylan F, O’Shea JJ, Gadina M. JAK inhibitors: Ten years after. *Eur J Immunol* (2021) 51:1615–1627. doi: 10.1002/eji.202048922

4.	Chikhoune L, Poggi C, Moreau J, Dubucquoi S, Hachulla E, Collet A, Launay D. JAK inhibitors (JAKi): Mechanisms of action and perspectives in systemic and autoimmune diseases. *La Revue de Médecine Interne* (2025) 46:89–106. doi: 10.1016/j.revmed.2024.10.452

5.	Sáez-Cirión A, Mamez A-C, Avettand-Fenoel V, Nabergoj M, Passaes C, Thoueille P, Decosterd L, Hentzien M, Perdomo-Celis F, Salgado M, et al. Sustained HIV remission after allogeneic hematopoietic stem cell transplantation with wild-type CCR5 donor cells. *Nat Med* (2024) 30:3544–3554. doi: 10.1038/s41591-024-03277-z

6.	Marconi VC, Moser C, Gavegnano C, Deeks SG, Lederman MM, Overton ET, Tsibris A, Hunt PW, Kantor A, Sekaly R-P, et al. Randomized Trial of Ruxolitinib in Antiretroviral-Treated Adults With Human Immunodeficiency Virus. *Clin Infect Dis* (2022) 74:95–104. doi: 10.1093/cid/ciab212

7.	Ezeonwumelu IJ, Garcia-Vidal E, Felip E, Cabrero-de Las Heras S, Clotet B, Badia R, Ballana E, Riveira-Muñoz E, Nevot M. Modulation of IRF7-driven transcription as a strategy to control HIV-1 latency. *Front Immunol* (2026) 17:1735192. doi: 10.3389/fimmu.2026.1735192

8.	Akcora BÖ, Dathathri E, Ortiz-Perez A, Gabriël AV, Storm G, Prakash J, Bansal R. TG101348, a selective JAK2 antagonist, ameliorates hepatic fibrogenesis in vivo. *FASEB J* (2019) 33:9466–9475. doi: 10.1096/fj.201900215RR

9.	Song Z, Liu X, Zhang W, Luo Y, Xiao H, Liu Y, Dai G, Hong J, Li A. Ruxolitinib suppresses liver fibrosis progression and accelerates fibrosis reversal via selectively targeting Janus kinase 1/2. *J Transl Med* (2022) 20:157. doi: 10.1186/s12967-022-03366-y

10.	Ely W. Randomized Double-Blind Placebo-Controlled Trial EValuating Baricitinib on PERSistent NEurologic and Cardiopulmonary Symptoms of Long COVID (REVERSE-LC). [Clinical trial registration]. clinicaltrials.gov. (2025). https://clinicaltrials.gov/study/NCT06631287 [Accessed July 29, 2025]

1. JAK inhibitors carry a black box warning label for increased risk of  serious heart-related events, cancer and death from the Food and Drug Administration (FDA).
