# Supplementary material for: Targeting the JAK/STAT pathway in chronic viral infections: opportunities and challenges for immunomodulation
Source: Front Immunol. 2026 Aug 21;17:1887876. doi: 10.3389/fimmu.2026.1887876 (PMC13542882; doi:10.3389/fimmu.2026.1887876)
Supplement: Supplementary file 3 [file Table2.docx]

Supplementary Material Table S2

| **Disease Association associated** | **Cause of chronic infection** | **Viral targets on JAK/STAT pathway** | **Viral compound involved** | **Mechanism** | **Consequences on antiviral defense** | **Possible implication for JAK-i outcomes** |
| --- | --- | --- | --- | --- | --- | --- |
| **HIV-1** (1,2) | | | | | |  |
| AIDS | Viral DNA integration into host genome | **Decrease STAT1 activation** | Vpu | Inhibition of **STAT1** | Compromised antiviral defense and promote viral persistence | Antiviral and anti-inflammatory effects |
|  |  |  | Nef | Unknown |  |  |
|  |  |  | Vif | **STAT1** proteasomal degradation |  |  |
|  |  |  | Tat | Unknown |  |  |
|  |  |  | Envelope proteins | Atypical and inefficient **STAT1** activation in T cells |  |  |
|  |  | **Increase  STAT1 activation** | Envelope proteins | Unknown | Hijack components of the host antiviral defense with upregulation of inflammatory genes, while attenuating their full efficacy |  |
|  |  |  | Tat | Interaction with specific gene promoters and transcription factors in antigen-presenting cells |  |  |
|  |  |  | Nef | Stimulation alpha and beta isoform of **STAT1** in monocyte-derived macrophages |  |  |
|  |  | **Decrease STAT3  activation** | Envelope proteins | HIV-1 DNA integration at the **STAT3** locus in primary human CD4+ T-cells | Immune dysregulation with IL-17 reduction, weaken host defenses to promote viral persistence |  |
|  |  | **Increase  STAT3 activation** | gp120 | Upregulation of IL-6 in a CCR5 dependent manner in dendritic cells | Defective function of dendritic cells to induce early immune dysfunction and viral immune evasion with increase of mRNA and protein expression of CCR5 and CCR2 |  |
|  |  | **Decrease STAT5 activation** | Envelope proteins | Dysregulation of **STAT5**, including the induction of a truncated isoform | Represses HIV-1 transcription in myeloid cells and monocytes contributes to viral latency |  |
|  |  | **Increase STAT5 activation** | Envelope proteins | Observed in lymphoid, monocytic and primary monocyte-derived macrophages | Impaired T cell survival and defective macrophage function, promotes HIV-1 transcription and enhance productive infection with increase of mRNA and protein expression of CCR5 and CCR2, and also higher levels of integrated proviral DNA in PWH |  |
|  |  | **Increase SOCS1 and SOCS3 expression** | Tat | Induces SOCS-2 expression in primary blood monocytes | Inhibits IFN-γ pathways and attenuates antiviral interferon responses |  |
|  |  |  | Unknown | Dysregulates endogenous **SOCS-1** and **SOCS-3** expression in host immune cells (dendritic cells, CD4⁺ T cells, lymphoid and mucosal tissues) | Promotes chronic immune activation while impairing effective antiviral immunity |  |
| **HTLV-1** (3) | | | | | |  |
| Adult T-cell leukemia |  | **Increase activation of JAK1, JAK3 and STAT5** | Tax | Tax interacts and transactivates two autocrine pathways of IL-2/IL-2Rα, IL-15/IL-15Rα and one paracrine pathway of IL-9 leading to the activation of the JAK/STAT pathway and phosphorylation of **STAT5** | Spontaneous proliferation of T-cells leukemic | Decrease of proliferation of cancer cells |
| **SARS-CoV-2** (4–6) | | | | | |  |
| COVID-19 and Long COVID (chronic inflammation and multi-organ damage) | Cytokine storm and multi-organ dysfunction | **Decrease STAT1 activation** | Nsp9 | Inhibits IFN-γ/α/β | Impairing JAK/STAT pathway activation to compromised antiviral defense and promote viral persistence | Antiviral and anti-inflammatory effects |
|  |  |  | Nsp5 | Induce autophagic degradation of **STAT1** |  |  |
|  |  |  | Nsp13 | Unknown |  |  |
|  |  |  | Nsp14 | Inhibit IFNAR or type I IFN for lyposomal degradation |  |  |
|  |  |  | Nsp1 | Acts on IFN-α signaling to inhibit **STAT1** phosphorylation |  |  |
|  |  |  | Nsp3 | Inhibit **STAT1** phosphorylation |  |  |
|  |  |  | Nsp6 | Unknown |  |  |
|  |  | **Decrease  STAT2 activation** | Nsp1 | Acts on IFN-α signaling to inhibit **STAT2** phosphorylation |  |  |
|  |  |  | Nsp3 | Inhibition **STAT2** phosphorylation |  |  |
|  |  |  | Nsp6 | Unknown |  |  |
|  |  |  | Nsp13 | Unknown |  |  |
|  |  | **Increase** **STAT3**  **activation** | N protein and Nsp5 | **STAT3** mediated IL-6 and IL-23 signaling to maintain Th17 cell differentiation and activate **STAT3**/JAK2 signaling | JAK/**STAT3** pathway increase, enhance IL-17 expression and facilitate NF-κB hyperactivation and abrogates IFN-y production. This triggers a self-sustaining IL-6 positive feedback loop, promoted by NF-kB that feeds the hyperinflammatory response with increased CCL2 levels |  |
|  |  | **Increase SOCS3 expression** | Unknown | Unknown | Reduce IFN effects and T cell-dependent B cell activation |  |
| **EBV** (7–12) | | | | | |  |
| Malignancies including: such as Burkitt’s lymphoma, Hodgkin’s disease and others, B cell lymphoma and other cancers | Latent infection program in resting memory B cells | **Increase STAT3 activation** | LMP1 | Interaction between the LMP1 consensus tumor-necrosis-factor-receptor-associated factor (TRAF)-binding domains and TNFR-associated factor 2 (TRAF2) and JAK3 | Upregulation of the expression of LMP1, creating a positive feedback and facilitate NF-κB hyperactivation, thus inducing inflammation to maintain proliferation and survival of EBV-infected B cells in latency | Decrease the latency/survival state of EBV |
|  |  |  | EBNA2 | via JAK3 interaction |  |  |
|  |  |  | LMP2A | Via PI3K/BTK pathway in B-cell |  |  |
|  |  | **Decrease STAT2 and STAT3  activation** | BGLF2 | Binds to Tyk2 to inhibits its phosphorylation | Block type I IFN signaling to promote EBV reactivation. Block expression of IFN-stimulated genes (ISGs) IRF1, IRF7, and MxA |  |
| **CMV** (13–18) | | | | | |  |
| Life threatening disease risk such as congenital birth anomalies, pneumonitis | Latency in myeloid lineages and hematopoietic progenitor cells | **Increase  STAT1 activation** | Latency protein UL138 | **STAT1** phosphorylation via interaction with the deubiquitinating complex, UAF1-USP1 | Recruitment of antiviral innate host responses for viral latency, limit its viral replication by using **STAT1** as a transcription factor to control UL138 expression and activate the viral major immediate early promoter (MIEP) | Impact the latency/reactivatiof CMV |
|  |  |  | Immediate-early 1 (IE1) protein | **STAT1** phosphorylation by removing **STAT3** from the cytoplasmic signaling pool for **JAK1** kinase. | Activation of **STAT1**-responsive genes |  |
|  |  | **Decrease STAT3  activation** | IE1 | Interaction with **STAT3** | Blocking host antiviral responses into nucleus to help viral replication |  |
|  |  |  | Unknown | Inhibition of **STAT3** phosphorylation but independent of IFNAR1, IL-10, IL-6 and JAK2 and restricted to MCMV-infected cells and not transmitted to bystander cells | Promotes DNA replication, and subset of IL-6/STAT3-regulated genes, including **SOCS3** are suppressed. |  |
|  |  | **Increase** **STAT3 activation** | Unknown | **STAT3** phosphorylation in HepG2 cells and primary human hepatocytes | Virus-induced phosphorylation blocks downstream signaling, disabling the antiviral response. This enhances HepG2 tumor growth and promotes primary hepatocyte transformation for HCC development |  |
|  |  |  | UL111A gene | Activation of JAK/**STAT3** pathway by cmvIL-10 binding to human cellular IL-10 receptor. cmvIL-10 is a viral homolog of IL-10 encoded by UL111A gene | Promotes transcription of immunosuppressive genes like SOCS3 |  |
|  |  |  | IE2 | Inhibits macrophage overactivation to raise IL-10 levels and thus activates the IL-10/**STAT3** signaling pathway | Inhibits macrophage antigen presentation to suppress T-cell responses. Immune evasion |  |
| **HSV-1** (5,19,20) | | | | | |  |
| Meningo-encephalitis (HSE especially in neonates and the elderly population) | Latency in a specific subset of sensory neurons, different from neuron-infected cells after viral reactivation | **Increase** **STAT3 activation** | Unknown | **STAT3** phosphorylation | Inhibition of HSV replication | Activation of HSV-1 possible |
|  |  | **Decrease JAK1 activation** | Ubiquitin-specific protease (UL36USP) | Binds to the IFNAR2 subunit and block the interaction between **JAK1** and IFNAR2 | Allows innate immune evasion by blocking type I IFN-mediated signaling |  |
|  |  | **Increase SOCS1 and SOCS3 expression** | γ134.5 gene | Recruits protein phosphatase 1α to dephosphorylate the alpha subunit of the translation initiation factor 2 (eIF-2α) | Evade the host immune response, suppressing interferon responses to prevent viral clearance which leads to persistent infection and chronic inflammation. |  |
| **HBV** (5,13,21–23) | | | | | |  |
| Long-term liver inflammation, fibrosis, hepatocarcinogenesis with hepatitis, cirrhosis, hepatocellular carcinoma (HCC) and liver cancer | Exploit the host’s SOCS-mediated feedback mechanisms to block JAK/STAT signaling, inhibit interferon responses, and establish chronic infection | **Decrease STAT1 activation** | HBV pol | Impaired **STAT1** phosphorylation It blocks the interaction of importin-α5, preventing STAT1/2 dimer nuclear translocation and ISG expression | Impairing JAK/STAT pathway activation to compromised antiviral defense to promote viral replication | Antiviral and anti-inflammatory effects |
|  |  |  | Unknown | Recruiting corepressors and interfering with **STAT1** binding to the cccDNA |  |  |
|  |  |  | core protein (p22) | Block nuclear import of **STAT1** |  |  |
|  |  |  | Unknown | Alter the epigenetic landscape to reduce the expression of **STAT1**. For example, **STAT1** methylation |  |  |
|  |  | **Increase** **STAT3 activation** | HBV X protein (HBx) | **STAT3** phosphorylation by activating the IL-6 signaling | Activated **STAT3** interacts with hepatocyte nuclear factor 3 (HNF-3) and different target genes, causing numerous effects on host and antiviral immunity, viral replication and HCC |  |
|  |  |  | hepatitis B surface antigen (HBsAg) | Unknown | Induce monocytes to become myeloid-derived suppressor cells to suppress the immune response and are involved in renal tubular epithelial cell apoptosis caused by hepatitis B virus (HBV) genotype G (HBVGN) |  |
|  |  | **Increase SOCS1 and SOCS3 expression** | HBV surface and antigens (HBsAg, HBeAg) | Unknown | Evade the host immune response, suppressing interferon responses to prevent viral clearance which leads to persistent infection and chronic inflammation |  |
| **HDV** (24) | | | | | |  |
| Hepatocellular carcinoma (HCC), most aggressive form of chronic viral hepatitis | Co-infection with HBV | **Increase JAK1 activation** | Unknown | Unknown | JAK1 has been shown to exhibit proviral activity *in vitro* during HDV infection, including primary human hepatocytes | Antiviral effects |
| **HCV** (5,13,22) | | | | | |  |
| Long-term liver inflammation, fibrosis, hepatocarcinogenesis with hepatitis, cirrhosis, hepatocellular carcinoma (HCC) and liver cancer | Exploit the host’s SOCS-mediated feedback mechanisms to block JAK/STAT signaling, inhibit interferon responses, and establish chronic infection | **Increase**  **STAT3 activation** | NS5A protein | Through the PI3K/Akt and IL-6 pathways | Induce oxidative stress and activates NF-κB, which helps HCV replicate by blocking the immune system and regulating genes that may cause HCC | Antiviral and anti-inflammatory effects |
|  |  |  | RNA NS4B, NS5B | Activates **STAT3** through oxidative stress | Induces the activation of tyrosine and MAP kinases, DNA damage via ROS |  |
|  |  |  | Core protein | Activate NANOG via **STAT3** to upregulate cylin-D1 expression | Enhanced cell proliferation and contribute to HCV-mediated pathogenesis by impairing the antiviral T-cell response |  |
|  |  |  | HCV RNA | In liver cells, bind and sequester miR-122, to avoid regulation **STAT3** expression | Induce upregulation **STAT3** expression and inhibit the expression of IFN-α/β, thus reducing innate immune system |  |
|  |  | **Increase SOCS1 and SOCS3 expression** | Unknown | Unknown | Evade the host immune response, which leads to persistent infection and chronic inflammation |  |

**Supplementary Table S2: Summary of JAK/STAT role and JAK-i data by virus.**

**Reference for Table S2:**

1.	Tolomeo M, Cascio A. The STAT Signaling Pathway in HIV-1 Infection: Roles and Dysregulation. *Int J Mol Sci* (2025) 26:9123. doi: 10.3390/ijms26189123

2.	Farjami Z, Akbarin MM, Álvarez HR. Suppressor of cytokine signaling (SOCS) proteins in human retroviral infections. *Mol Biol Rep* (2026) 53:366. doi: 10.1007/s11033-026-11513-7

3.	Daenthanasanmak A, Lin Y, Zhang M, Bryant BR, Petrus MN, Bamford RN, Thomas CJ, Miljkovic MD, Conlon KC, Waldmann TA. Enhanced efficacy of JAK1 inhibitor with mTORC1/C2 targeting in smoldering/chronic adult T cell leukemia. *Transl Oncol* (2021) 14:100913. doi: 10.1016/j.tranon.2020.100913

4.	Lei S, Chen X, Wu J, Duan X, Men K. Small molecules in the treatment of COVID-19. *Signal Transduct Target Ther* (2022) 7:387. doi: 10.1038/s41392-022-01249-8

5.	Low ZY, Wen Yip AJ, Chow VTK, Lal SK. The Suppressor of Cytokine Signalling family of proteins and their potential impact on COVID‐19 disease progression. *Rev Med Virol* (2022) 32:e2300. doi: 10.1002/rmv.2300

6.	Attiq A, Afzal S, Wahab H, Ahmad W, Kandeel M, Almofti Y, Alameen A, Wu YS. Cytokine Storm-Induced Thyroid Dysfunction in COVID-19: Insights into Pathogenesis and Therapeutic Approaches. *Drug Des Devel Ther* (2024) Volume 18:4215–4240. doi: 10.2147/DDDT.S475005

7.	Murata T, Sugimoto A, Inagaki T, Yanagi Y, Watanabe T, Sato Y, Kimura H. Molecular Basis of Epstein–Barr Virus Latency Establishment and Lytic Reactivation. *Viruses* (2021) 13:2344. doi: 10.3390/v13122344

8.	Thorley-Lawson DA. Epstein-Barr virus: exploiting the immune system. *Nat Rev Immunol* (2001) 1:75–82. doi: 10.1038/35095584

9.	Li W, Zhuang Y, Shao S-J, Trivedi P, Zheng B, Huang G-L, He Z, Zhang X. Essential contribution of the JAK/STAT pathway to carcinogenesis, lytic infection of herpesviruses and pathogenesis of COVID‑19 (Review). *Mol Med Rep* (2024) 29:39. doi: 10.3892/mmr.2024.13163

10.	Chen H, Hutt-Fletcher L, Cao L, Hayward SD. A Positive Autoregulatory Loop of LMP1 Expression and STAT Activation in Epithelial Cells Latently Infected with Epstein-Barr Virus. *J Virol* (2003) 77:4139–4148. doi: 10.1128/JVI.77.7.4139-4148.2003

11.	Konforte D, Simard N, Paige CJ. Interleukin-21 regulates expression of key Epstein-Barr virus oncoproteins, EBNA2 and LMP1, in infected human B cells. *Virology* (2008) 374:100–113. doi: 10.1016/j.virol.2007.12.027

12.	Liu X, Sadaoka T, Krogmann T, Cohen JI. Epstein-Barr Virus (EBV) Tegument Protein BGLF2 Suppresses Type I Interferon Signaling To Promote EBV Reactivation. *J Virol* (2020) 94:e00258-20. doi: 10.1128/JVI.00258-20

13.	Chang Z, Wang Y, Zhou X, Long J-E. STAT3 Roles in Viral Infection: Antiviral or Proviral? *Future Virol* (2018) 13:557–574. doi: 10.2217/fvl-2018-0033

14.	Zarrella K, Longmire P, Zeltzer S, Collins-McMillen D, Hancock M, Buehler J, Reitsma JM, Terhune SS, Nelson JA, Goodrum F. Human cytomegalovirus UL138 interaction with USP1 activates STAT1 in infection. *PLOS Pathog* (2023) 19:e1011185. doi: 10.1371/journal.ppat.1011185

15.	Trilling M, Le VTK, Rashidi-Alavijeh J, Katschinski B, Scheller J, Rose-John S, Androsiac GE, Jonjić S, Poli V, Pfeffer K, et al. “Activated” STAT Proteins: A Paradoxical Consequence of Inhibited JAK-STAT Signaling in Cytomegalovirus-Infected Cells. *J Immunol* (2014) 192:447–458. doi: 10.4049/jimmunol.1203516

16.	Satani KH, Spencer JV. Human cytomegalovirus interleukin 10 (cmvIL-10) orchestrates crosstalk between the cellular IL-10 receptor and CXCR4. *Virology* (2025) 612:110693. doi: 10.1016/j.virol.2025.110693

17.	Zhang X, Wang Q, Han S, Song G, Wang B, Wang Y. Human cytomegalovirus-IE2 suppresses antigen presentation of macrophage through the IL10/STAT3 signalling pathway in transgenic mouse. *PloS One* (2025) 20:e0322334. doi: 10.1371/journal.pone.0322334

18.	Harwardt T, Lukas S, Zenger M, Reitberger T, Danzer D, Übner T, Munday DC, Nevels M, Paulus C. Human Cytomegalovirus Immediate-Early 1 Protein Rewires Upstream STAT3 to Downstream STAT1 Signaling Switching an IL6-Type to an IFNγ-Like Response. *PLoS Pathog* (2016) 12:e1005748. doi: 10.1371/journal.ppat.1005748

19.	Cao S, Zhou M, Ji S, Ma D, Zhu S. Recent Advances in the Study of Alphaherpesvirus Latency and Reactivation: Novel Guidance for the Design of Herpesvirus Live Vector Vaccines. *Pathogens* (2024) 13:779. doi: 10.3390/pathogens13090779

20.	Yuan H, You J, You H, Zheng C. Herpes Simplex Virus 1 UL36USP Antagonizes Type I Interferon-Mediated Antiviral Innate Immunity. *J Virol* (2018) 92:e01161-18. doi: 10.1128/JVI.01161-18

21.	Lei Z, Wang L, Gao H, Guo S, Kang X, Yuan J, Lv Z, Jiang Y, Yi J, Chen Z, et al. Mechanisms underlying the compromised clinical efficacy of interferon in clearing HBV. *Virol J* (2024) 21:314. doi: 10.1186/s12985-024-02589-3

22.	Xie J, Wang M, Cheng A, Jia R, Zhu D, Liu M, Chen S, Zhao X, Yang Q, Wu Y, et al. The role of SOCS proteins in the development of virus- induced hepatocellular carcinoma. *Virol J* (2021) 18:74. doi: 10.1186/s12985-021-01544-w

23.	Xu B, Tang B, Wei J. Role of STAT1 in the resistance of HBV to IFN‑α. *Exp Ther Med* (2021) 21:550. doi: 10.3892/etm.2021.9982

24.	Heuschkel MJ, Bach C, Meiss-Heydmann L, Gerges E, Felli E, Giannone F, Pessaux P, Schuster C, Lucifora J, Baumert TF, et al. JAK1 promotes HDV replication and is a potential target for antiviral therapy. *J Hepatol* (2024) 80:220–231. doi: 10.1016/j.jhep.2023.10.030
